# Case study analysis of end of life care development in the Chinese cultural context of Macao: a social movement perspective

**DOI:** 10.1186/s12904-021-00807-1

**Published:** 2021-07-09

**Authors:** Kuai In Tam, Elaine Haycock-Stuart, Sarah J. Rhynas

**Affiliations:** 1 Kiang Wu Nursing College of Macau, Est. Repouso No. 35, R/C, Macao, SAR PR China; 2 Nursing Studies, School of Health in Social Science, Teviot Place, the University of Edinburgh, Room 2M3, Doorway 6, Edinburgh, EH8 9AG UK; 3 Nursing Studies, School of Health in Social Science, the University of Edinburgh, Room 1.12, 18 Buccleuch Place, EH8 8LN Edinburgh, UK

**Keywords:** The modern hospice movement, Hospice care, Palliative care, End of life care, Chinese culture

## Abstract

**Background:**

The modern hospice movement is often recognised as a social movement. However, such understanding is primarily based on historic reflection and this approach has lacked theoretical exploration. There is a lack of systematic examination of the modern hospice movement by way of social movement theories.

**Aim:**

Focusing on the Chinese socio-cultural context of Macao, this study aimed to understand the EoLC movement by applying the social movement theory, the Framing Perspective, as proposed by Snow and Benford in 1988.

**Methods:**

A case study approach was conducted. Semi-structured interviews were held between 2012 and 2013, with pioneers (n = 11) of the EoLC in Macao. Thematic analysis was adopted to analyse the interviews.

**Results:**

The Framing Perspective analysis illuminated that there was both growth and stagnation of the EoLC movement. Three themes emerged: 1) the suffering of people at the end of their lives was considered as a social problem needed to be addressed urgently, 2) the incoherent EoLC strategies developed by pioneers indicated the lack of internal ideological cohesion within the movement, 3) external constraints contributed to the stagnation of the movement.

**Conclusions:**

The EoLC development in Macao can be understood as a social movement. The Framing Perspective provided a theoretical way to understand the emergence of EoLC; offering a novel perspective to conceptualise the modern hospice movement. This sociological and theoretical lens opened up new ways for future research to study the emergence of EoLC in different socio-cultural contexts.

**Supplementary Information:**

The online version contains supplementary material available at 10.1186/s12904-021-00807-1.

## Background

While the modern hospice movement is acknowledged and referred to as a social movement [1], there is a lack of exploration of the emergence of end of life care (EoLC] from a theoretical, social movement perspective. The conceptualisation of the modern hospice movement mainly concerns the establishment of the dissemination of the principles of hospice care, and the establishment of hospices and related EoLC services [2–7]. Several studies have investigated the development of EoLC in countries with a range of different socio-cultural backgrounds [8–13]. However, most available literature discussed issues pertinent to the practical and conceptual introduction of EoLC [8, 11–24] and there is a notable lack of discussion on the theoretical underpinning of EoLC initiatives. While studies by Hodges and Read [25] and Elsey [26] have utilised theoretical constructs to examine the hospice movement in the United Kingdom and South Australia, the focus of their studies was on the institutionalisation of hospice care within the area of cancer care [25], and the increasing secularisation of the hospice movement as it has been driven by professionals, establishing hospice and palliative care as a knowledge-based discipline [26]. To a certain extent, these studies have offered a theoretical examination of the hospice movement, however, they primarily examined the institutional structure of hospice care, and the broader shift of the hospice movement to a professionally driven discipline. There is still a lack of systematic examination of the emergence of EoLC as a social movement, especially by way of a social movement theory. Targeting this knowledge gap, this study has adopted a social movement theory, the Framing Perspective, to examine data to analyse the emergence and stagnation of EoLC movement in the Chines socio-cultural context of Macao.

### End of life care in Macao

The beginning of Macao’s EoLC was marked by the establishment of the Association of Friends of Charity of Macao (AFCM) in 1996, offering free health education, counselling and financial advice to those affected by cancer [27]. The year 2000 marked the establishment of a community home visit service, the Peace and Hope Centre, and the first hospice, the Hospice and Palliative Care Centre (HPCC). The Peace and Hope Centre provided non-clinical home visits, encompassing spiritual (Christian-based) and psychological support for people affected by terminal cancer [28]. HPCC remained the only clinical palliative and EoLC service in Macao until 2019, when the Macao government hospital established an inpatient palliative care ward. Reflecting on the EoLC development in Macao, it has a substantial time gap (19 years) between the establishment of the first and last clinical service; furthermore, there are still no records of any professional bodies being devoted to aspects of EoLC in Macao, nor any legislation or professional guidelines specifically relevant to EoLC related issues. Macao was not included in the quality of death index published by the Economist Intelligence Unit [29] and no assessment has thus far been found that dealt with an evaluation of the quality of EoLC offered in Macao.

The current study therefore aimed to address the lack of empirical evidence on the analysis of the modern hospice movement by way of any theoretical perspective informed by, or based upon, the concept of social movement, and the lack of research investigating the establishment of EoLC by using the perspective of social movement in the socio-cultural context of Macao. Integrating the two enquiries, the study aimed to understand the EoLC movement within the Chinese socio-cultural context of Macao, by applying the social movement theory, the Framing Perspective.

## Methods

### Study design

The qualitative case study model proffered by Robert Stake [30–32] was adopted in examining the emergence of EoLC in Macao, and developing a theoretical understanding of the EoLC establishment from a social movement perspective. The *‘EoLC within the context of Macao’* was determined as ‘the case’, in order to reinforce the focus of enquiry for the present study.

### Participants and recruitment

Participants recruited for this study were pioneers, including initiators, developers and nurse educators, who had been substantially involved in the establishment of EoLC in Macao. For the individuals who initiated the concept and the service of EoLC in Macao were identified as initiators/pioneers; for the individuals involved in the subsequent development of EoLC in Macao were identified as developers. Both initiators and developers were considered as the people best able to give insights to the phenomenon of the study. Inclusion of nurse educators was mainly because they were found to have substantially involved in introducing and developing EoLC education in Macao [33–35]. Participants of this study were therefore recruited from: i) the inpatient hospice, ii) the community EoLC service and iii) a nursing education sector, where Macao’s public promotion of the concept of EoLC and the introduction of palliative and EoLC first began. Six participants (two initiators, three nurse educators and a hospice developer) were approached and recruited for this study by way of purposive sampling. Five more potential participants were identified by snowball sampling. A total of 11 participants were recruited, with verbal and written informed consent being obtained from all participants.

### Data collection

Semi-structured, in-depth interviews were conducted with the participants. In keeping the quality and relevance of the materials generated through interviews [30, 36], a semi-structured interview guide with five open-ended questions oriented to the research aim, guiding the interview process and keeping focus on the subject was developed prior to the interview process. The open-ended questions were developed to allow the researcher to learn about participants’ professional backgrounds, to understand their experiences and interpretations regarding end of life (EoL) issues, and to learn about the trigger/motivation in pioneering/developing EoLC in Macao. The interview guide was developed in both Chinese and English (please refer to supplementary material for the English interview guide-Additional file 1), allowing the research to include participants with different language backgrounds. Whilst the interview guide kept the researcher focused on the research enquiry, the researcher used it only as a guide during each interview so allowing her to prompt and further explore new topics that emerged during the interviews. All but one of the interviews were conducted in Chinese, one interview was conducted in English. All interviews were transcribed verbatim. In order to maximise the validity, reliability and quality of data analysis, as suggested by Twinn [37], the researcher decided to base the data analysis on the Chinese transcripts. The analysis of the only English interview was conducted in English. Only interview segments of the Chinese transcriptions relevant to data analysis were translated into English for the purpose of presenting the research results. The researcher had consulted a bilingual colleague to crosscheck a few translated excerpts to ensure the accuracy of the translation [38]; the colleague confirmed translation to be accurate.

### Data analysis

A thematic analysis approach [39] was adopted to structure the analytical process for the study. The process of data immersion during analysis facilitated the researcher to determine that, the Framing Perspective [40–42] was a suitable theoretical framework to examine the emergence of the EoLC in Macao, and to aid the development of a theoretical understanding of the EoLC movement in Macao. The computer-assisted data management software (QSR NVivo version 11) was used to manage and facilitate the analysis of the collected data.

### Establishing the theoretical context of the study: the Framing Perspective

The Framing Perspective was first proposed by Snow, Rochford, Worden and Benford in their 1986 article: “Frame alignment processes, micromobilisation, and movement participation” [40]. In the article, the concept of ‘framing’ is presented as a theoretical extension of the work of frame analysis proposed by Erving Goffman in 1974, with a focus on the study of social movements [42]. Theorised by Goffman, a ‘frame’ is the definition of a situation one becomes subjectively involved in, signifying ‘a lore of understanding’, ‘an approach’ and ‘a perspective’ (p. 21) [43]. Further, the process of framing is a channel for individuals to ‘locate, perceive, identify and label’ (p. 21) [43] their experiences. Adopting the conceptualisation of framing as offered by Goffman, in the study of social movement, framing denotes an active and dynamic process of meaning construction; with such construction evolving on a constant basis [40, 41, 44]. Further developed from Goffman’s theory, Snow et al. [40] proposed the four frame alignment processes in the study of social movements: frame bridging, amplification, extension and transformation. These four alignment processes formed the foundation of the theoretical construct of the Framing Perspective on social movements suggested by Snow, Rochford, Worden and Benford [40]. Explained by these authors, the purpose for establishing the Framing Perspective was to address the lack of theoretical apparatus in the field of social movement study, by elucidating the process of movement recruitment, participation and conversion; i.e. the frame alignment processes [42].

In addition to the four frame alignment processes, Snow & Benford [41] also introduced the concept of three core framing tasks encompassing diagnostic framing, prognostic framing and motivational framing. The frame(s) constructed in these three framing tasks are those adhered to by social movement participants; stemming from these frames are actions in promoting frame alignment amongst wider audiences.

Taking into consideration of the lack of examination of the emergence of EoLC as a social movement, the researcher decided to consider a theoretical construct that would fit into the research enquiry, and to develop a theoretical understanding of EoLC as a social movement. After a comprehensive literature review relating to studies of social movement, the researcher encountered the Framing Perspective. Having explored the conceptual elements of the Framing Perspective (as explained above), this perspective reflected many of the features of the EoLC initiatives and programmes in Macao. The researcher therefore decided that the Framing Perspective would provide the theoretical underpinning to aid the interpretation of initiators and developers in the process of developing EoLC in Macao whilst, at the same time, facilitating the articulation of a theoretical understanding of the EoLC in Macao.

### Reflexivity

Reflexivity of the researcher in this study was informed by the position as an ‘insider’ as well as an ‘outsider’. The recognition of this joint researcher position illuminated the researcher’s reflexivity during the research process, in that the researcher was aware of how her experiences, or lack of them, might contribute to the construction of meanings, from structuring research questions, facilitating interviews, to handling, interpreting and analysing the collected research data [45, 46].

### Rigour

The current study had adopted the position of quality assessment for case study, advised by Thomas [47], to ensure the quality and robustness of this research. The researcher had kept clear documentation with regards to every step taken in conducting this study. The study had also provided participants with their interview transcripts, allowing them to confirm their responses and to make amendments should they see the need to do so. In offering this option to the participants, the study intended to safeguard participants’ confidentiality, but also wished to ensure the quality of the data [48].

## Results

### Problem conceptualisation: the suffering of people as experienced at the end of their lives

The rise of a social movement usually intends to change a problematic situation (the grievance) identified by the movement’s adherents [40, 41, 44]. The rise of the EoLC movement in Macao, was found to have stemmed from one particularly problematic situation identified by the initiators of Macao, that was the suffering people experienced at the end of their lives. According to the Framing Perspective, the consensus of a problematic situation is often attained without complication, the attributional consensus, which is the consensus of the diagnostic frames, is usually more complicated [41]. While the study found that the three initiators of the ‘EoLC in Macao’ having different personal and professional experiences related to EoL situations, they had reached consensus and conceptualised the same problem. However, the attribution of the problem was found to be different amongst the three initiators. For instance, the hospice initiator attributed the problem to the absence of clinical EoLC, while the community EoLC initiator ascribed the problem to the absence of community EoLC in addressing the abandonment of the dying; for the nursing education initiator, her own suffering caused by the separation of life from death had become her motivation. The diversity of these attributions had led to the construction of various solutions (prognostic frames) to target the problem. As a consequence, the EoLC movement in Macao was being driven in different developmental directions.

With respect to the hospice initiator, introduction of the hospice service in Macao was inspired by initiator’s early visits to EoLC services in Hong Kong (HK) and Taiwan.

*“Through the Nursing Association of Macao, I have encountered nursing associations in other areas. So I knew about HK and Taiwan those areas... places further away I didn’t know too much, because at that early time, our association didn’t contact associations in other areas. However, we still knew about Taiwan and HK, in particular, HK’s Bradbury Hospice, this was very famous when it first established. So I knew about this type of end of life nursing.”(Hospice Initiator)*

Learning from her encounters with the dying, the hospice initiator realised there was a substantial care gap between acute illnesses and chronic incurable conditions in the healthcare system in Macao.

*“I have also encountered some cancer patients would have nowhere to live at their terminal stage. One patient rented a shipping container, and in there was where the patient slept and planned to endure sufferings through his last phase of life. Because the patient didn’t think he could stay at home because his family were not able to manage. Perhaps, the family situation was not able to offer anything. So in fact, cancer patients at those times really suffered a lot during their final stage.”(Hospice Initiator)*

The hospice initiator noticed that death and dying was not the priority of mainstream healthcare system, prompting a lack of institutional support offered to people suffering from EoL issues. The introduction of the hospice in Macao was therefore, the initiator’s effort in addressing the care gap between acute and EoLC. The establishment of the hospice was the prognostic approach in addressing the suffering people experienced at the end of their lives.

For the nursing education initiator, she first learned the concept of EoLC from a nursing colleague from HK.

*“In 86, 87, I further studied in HK and met some nursing colleagues there. I remember there was one colleague, she worked in a hospice service in the hospital. She started that hospice service. She has studied at the origin of (hospice care) in England. After her studies, she came back to HK and started this service. I remembered it deeply and was very interested. I still remember her face. After talking with her and learned some ideas, I wrote more articles. The Nursing Association at the time had a journal column and I wrote quite a few articles about end of life issues, some related to bioethics and organ transplantation. I have also organised a group of people to visit the Bradbury Hospice in Hong Kong and wrote more about the experience there too.” (Nursing educator initiator)*

In deliberating over her professional background and ability, the nursing education initiator decided that the construction of the EoLC concept would be more efficient and fitting if oriented towards the field of nursing. Furthermore, the initiator openly discussed about the pain and heartache she experienced.

*“It is all because I have experienced it now, I have the feeling now. In cancer, it doesn’t just affect the patient but the entire family. This is my experience. Because myself, my mother-in-law and my husband, we all had to take care of my father-in-law. (…) I feel it is very important to help relatives of the dying to go through difficulties. This is something we have to do. This is my feeling. That is something we have to push forward, because I have experienced it personally.” (Nursing educator initiator)*

Depicting the journey of her father-in-law’s death, the initiator understood the hardship in caring for a dying person, the practical difficulties and emotional distress during the process. The interpretation of the nursing education initiator was based on her personal pain as a result of death. After experiencing her father-in-law’s death, the initiator realised the pain death and dying could bring to a family. For the nursing education initiator, the personal experience of death and dying, and her subsequent encounter with the HK colleague had laid the foundation in respect to her involvement in the EoLC movement in Macao. The conceptualisation of the prognostic frames and approaches of the nursing education initiator was primarily grounded in this interpretive background of hers.

For the community EoLC initiator, the background as a physician from the United States had influenced the initiator’s perspective in relation to the EoLC movement in Macao. Working in a community clinic, the initiator had witnessed a need for EoLC care in the community.

*“As I mentioned, one of the reasons for going specifically into terminal care ministry was that it was something that particularly the government health services had not yet focused on. And so this was recommendation of our nurses, whereas previously were providing more general community nurse service and its compared to the population it was very small amount of people we were able to reach but the model was established and then, when the government then, also adopted this model as a part of their healthcare provisions, then we, our nurses recommended we focused more on terminal illness at that time.” (Community EoLC initiator)*

Emerged from the data, the various solutions developed by initiators corresponded with the different diagnostic frames conceived, and the different prognostic frames and approaches developed; a phenomenon explained by the Framing Perspective as a result of diagnostic framing is often projected onto prognostic framing, leading to different solutions developed by social movement initiators. Table 1 below summarises the various diagnostic and prognostic frames conceptualised by the three EoL initiators in Macao.

**Table 1:** Diagnostic and prognostic frames conceptualised within the end of life care movement in Macao

	Diagnostic Frames	Prognostic Frames	Prognostic Approaches
Hospice Initiator	Absence of clinical EoLC	To alleviate suffering at the end of life	Establishing the first hospice
Community EoLC Initiator	Abandonment of the dying Absence of community EoLC to address abandonment of the dying	To alleviate suffering at the end of life To promote acceptance of death	Establishing the first EoLC home visit service
Nursing Education Initiator	Personal encounter of EoL experience Separation of life from death in nursing education	To promote acceptance of death	Constructing the EoLC concept in the nursing field and public sphere

Regarding the establishment of EoLC in Macao, whilst the articulation of frames, by EoLC initiators, was based on the shared grievance, the meanings rendered to those frames were diverse. This diversity was particularly visible amongst the prognostic frames, thereby contributing to the incoherence of internal frames in the EoLC movement of Macao.

### Diversified solutions: intra-movement frame disputes

Suggested by Snow and Benford [41], an intra-movement frame dispute is the fragmentation of goals and/or strategies amongst different individuals/ groups, within one movement. Intra-movement conflict was observed in the EoLC in Macao. Whilst initiators shared a unified vision of the grievance, the problems they framed contributed to this grievance differed.

The establishment of the hospice in Macao was essentially grounded in the hospice initiator’s moral obligation to help those who suffer from terminal illnesses; the establishment of an inpatient unit was the key solution for addressing the absence of care for the dying. Specifically, the hospice initiator suggested the hospice would provide an alternative option, besides curative therapy.

*“It showed a real demand for end of life care. So at that time, we reported the demand to the government, to the Health Bureau, hoping that Macao would have this service in the future, because everyone else has it already. So we have suggested many times, we have written formal letters to the government this kind of thing. But as always, as you know, acute emergency matters take priority in Macao, so the starting of the service was not that easy.”(Hospice initiator)*

Despite the slow progress in developing EoLC and the systemic priority placed on acute emergency care, the hospice initiator continued to push forward the establishment of the hospice.

Focusing on promoting and constructing the EoL concept amongst the general population and healthcare professionals, the nursing education initiator pursued her effort in organising public workshops, however, the quote below showed that her prognostic approach was being challenged by people’s rejection regarding death.

*“Not accepting. Last year, I have invited Professor X (an EoLC expert) to promote this matter. Despite the professor was highly spirited, there were only a small amount of people responded. (…) For people from different fields, we have organised different seminars and workshops, but the response was not enthusiastic. Perhaps people think there are more important things than death and dying. (…) “When will we talk about life and death? It is nothing to do with me” Or “Death is so far away from me”.” (Nursing education initiator)*

The different cultural and professional backgrounds guiding each of these three initiators formed the basis of the interpretations for their experiences in relation to death and dying. The diversity of their backgrounds had led to the construction of various prognostic frames. The lack of collectiveness of the EoLC movement in Macao was supported by initiators not being aware of, and not exploring other agencies in implementing EoLC. As a result, the solutions remained segregated from one another, as the community EoLC initiator aptly demonstrated in the quote below.

*“Not aware of any that were specially related to that. Now in those years we were pretty internally focused, so we didn’t uh, not allow to have medical privileges at any of the hospitals in Macao. So we weren’t really connected with the other medical providers very well. Fortunately that’s changed, but. and so uh, but we were not aware of uh this service being available anywhere.” (Community EoLC initiator)*

Not only was the initiator unaware of existing organisations, but he also had limited external collaborations with other organisations. The lack of collectiveness—the lack of internal frame cohesion—implies that the EoLC in Macao was unable to garner support in supporting one movement, resulting the fundamental lack of cohesion across key parties of the movement. Findings of this study support the phenomenon of Macao in which the growth of EoLC had become stagnant after the initial wave of establishment.

Continuing the discussion on frame misalignment by way of the Framing Perspective, the section below further elaborates on the influence of the chain effect on the issue of a lack of external frame cohesion. This deficit results from the lack of consideration initiators had paid to the cultural divergence between the EoLC they proposed and the existing cultural context of Macao.

### The lack of external frame cohesion

In the data analysis, the study found that there had been a pervading cultural norm in Macao to maintain life at all cost and that this particular belief was in contradiction to the ethos of EoLC proposed by the initiators. Interpreting through the Framing Perspective, this contradiction of value demonstrates the difference between the prognostic frames proffered to address the suffering at the EoL in Macao, and the cultural insistence of sustaining a person’s life, regardless of quality. The conflicting nature between EoLC and the extant cultural element of life preservation had challenged the introduction of EoLC from the start, mainly because the ideologies conceptualised by initiators of EoLC were in complete contrast with the cultural beliefs held by the public of Macao.

*“… They feel that no resuscitation equals giving up on life. Yes, so when the patient is not for resuscitation, relatives will feel they are sending the patient to a place with no medical treatment. Relatives will find it very difficult to see past their obligations. They have not really understood the service of palliative care. So their thoughts are normal.” (Hospice developer)*

Evident in the above quote is a clear correlation between cultural expectations of sustaining life through resuscitation. Within the context of EoLC, a link was established between the idea of no treatment and no resuscitation; as a result, EoLC was being conceptualised as a way of abandoning the dying relatives. These findings lend support to existing research that, amongst Chinese patients and families, making a decision for the complete withdrawal of resuscitative measures was deemed unacceptable, due to the existing filial obligations; as a result, basic life support medications were still commonly given regardless of resuscitation status [49, 50]. End of life care was being conceptualised/perceived as a form of abandonment of the dying, contrasting the external cultural beliefs held by the people in Macao. The formation of this misconception is, as seen through the prism of moral liability, essentially embracing the opposite values to those of curative care. Findings of the current study show that most EoLC misconceptions stemming from the opposite values of curative care, are related to the context of inpatient EoLC.

*“Also, they didn't want to feel as if they have now come to the end and have to go to (the hospice) at the last moment of their lives. So, for them, they would perhaps choose to go to the hospital ward. Some people have asked to be transferred back to the hospital ward after coming into the hospice.” (Nursing education developer)*

As the above extract suggests, EoLC is conceptualised as a place where death is certain, whereas curative care is the more hopeful alternative. Furthermore, EoLC is also being seen as a failure, as illustrated by the quote below; hence, some people may choose to go back to curative care.

*They reject the hospice and what it has to offer. As I said before, they see that by going into the hospice, it symbolises failure. Complete utter failure and by that it means accepting death.” (Community EoLC developer)*

The rejection of EoLC primarily stems from the idea that the acceptance of EoLC symbolises the acceptance of death. The absence of cure and the absence of the intent to cure mean that EoLC does not align with the Chinese cultural desire and expectation of life preservation. Findings of the study show that the proposed EoLC in Macao was predisposed to both intrinsic and extrinsic contradiction from the moment it was first introduced. Intrinsically, the EoLC principle of ‘not striving for a cure at any cost’ is evidently incompatible with a culture that insists on life preservation; a conflict which has led to the development of misinterpretations. The resulting misinterpretations of EoLC, and their strong association with the certainty of death, have further reinforced the misalignment between EoLC and the existing cultural context, which has further inhibited the growth of the EoLC movement in Macao. However, in the process of analysis, the intrinsic contradiction between EoLC and the existing cultural attitudes regarding life preservation through curative measures had already been recognised by the initiators at the early stage when EoLC was first introduced.

The fact that EoLC is not able to offer life sustaining measures makes it intrinsically difficult to align with Macao’s existing cultural values. As display in the quote below, the introduction of EoLC was hindered by this internal contradiction between EoLC and the extant cultural values of sustaining life, which then contributed to systemic inhibition imposed directly on the development of EoLC in Macao.

*“We felt there was a real demand for end of life care. So, at that time, we had communicated the demand to the government and the Serviços de Saúde (The Health Department). We hoped Macao could have this service in the future because other places already had this service. So we had suggested many times, we wrote letters, this kind of thing. But as you know, acute matters needed to be attended first, so it wasn’t easy to start end of life care.” (Hospice initiator)*

The priority of the government was on life sustaining measures, a situation initiators were well aware of. In spite of such a challenge, initiators persisted with their beliefs and pioneered EoLC in their individual areas (despite they did not work together). However, as the study’s findings suggest, the problem of misalignment between EoLC and the existing culture regarding life preservation, had persisted. According to the Framing Perspective, the more compatibility that exists between the proffered frames of a social movement and the existing cultural narrations, the more acceptable and popular the movement becomes [51]. In Macao, the connection of EoLC and no resuscitation was therefore unable to align with the existing cultural expectations of life preservation, thereby inhibiting the acceptance of EoLC and the overall development of the EoLC movement.

Put forward by Benford and Snow [44], the frame alignment processes are strategies adopted to help social movement to negotiate the acceptance of frames in the extant cultural context which the movement is situated; these processes are involved strategically, as social movement begins to establish frames, and potentially enhance movement efficacy from the start. With respect to the initiation of EoLC in Macao, initiators had not involved any of the framing processes in generating frames at the beginning stage of the initiative. The frames underpinned the initiation of EoLC were predominantly grounded in the three initiators’ interpretations of the degree of a person’s suffering experienced at the EoL. Initiators did not contemplate the extant cultural element, the desire and expectation to preserve life, when developing the initial frames for the EoLC movement. This misalignment has contributed to the misinterpretation and misconceptualisation of EoLC, which then negatively affected the overall acceptance of the EoLC movement.

## Discussion

The Framing Perspective has: i) enabled the theorisation of intra-movement frame disputes, and ii) explained why the EoLC in Macao failed to meet the criteria of being cohesive and collective in a social movement. Despite the theoretical stance of the Framing Perspective has offered a degree of explanation regarding the intra-movement frame disputes of the EoLC movement in Macao, it is not able to address the specific concern of frame fragmentation within one movement. Despite possible structural differences, social movements should have “cohesion and continuity over time” (p. 24) [52].

Albeit the Framing Perspective depicted the lack of internal frame cohesion due to the discrepancy amongst the EoLC initiators, the theory was unable to fully facilitate the investigation of the emergence of frame disputes within the EoLC movement of Macao. In particular, problems remain regarding: a) how initiators came to devise incoherent strategies and b) how the diversity had continued without any observable collaboration between initiators. Pertaining to the deviated integration between the EoLC movement and the Chinese socio-cultural context of Macao, the Framing Perspective offered little consideration of the cultural component in frame articulation, and how this may potentially affect the development of social movements. The EoLC movement of Macao was based exclusively on the initiator’s ideologies in targeting a specific problem, also identified by the initiators, was not specifically discussed in the frame alignment processes in the Framing Perspective. However, as asserted by Benford [53] in his study on frame disputes in the nuclear disarmament movement, for most cases of social movements there are usually challenges inhibiting movement initiators to construct and impose a particular version of reality. It is unlikely that the framing processes would not have encountered any form of challenges on social movement introduction. In contrast to Benford’s [53] assertion, Macao’s EoLC was discovered to have been framed exclusively on and informed by the three initiators’ version of reality; in summary, they believed the suffering at the EoL should be alleviated through the means of EoLC. As findings of the research show, the development of the EoLC movement in Macao was subsequently hindered by the misalignment between the frames introduced by the movement and the belief in life preservation existing in the cultural context.

In the studies of social movements, it is well established that one of the biggest challenges for social movement initiators is the promotion of frames, that are often established in contradiction to the dominant culture, in which the movement is embedded [54]. In the case of EoLC in Macao, complete alignment between extant cultural values, and frames proffered by EoLC, could be difficult to achieve due to the substantial cultural divergence between the two variables. Nevertheless, the issue of frame misalignment is, in one way or another, attributed and heightened by the negligence of initiators to take into account this cultural divergence, when in the beginning stage of frame articulation for the EoLC in the case of Macao.

The EoLC in Macao epitomises a type of social movement that is different from the types suggested by Snow and Benford [41], Snow et al. [42] and Snow et al. [40], in that the current structure of framing processes informed by the Framing Perspective only offers a limited understanding of social movements that are developed via frames that are based on the version of reality in accordance with their initiators’ interpretations. The study does not argue that the construction of this ‘alternate reality’(p.679) [53], is impossible; in fact, this reality has already been constructed as is evident from the establishment of both the hospice and the community EoLC services in Macao. The findings of the lack of both internal and external frame cohesion demonstrate how these factors have impacted the EoLC movement, and are thereby contributing to the theoretical understanding of such care model’s developmental process. Given the example of the EoLC movement in Macao, the study argued that the understanding of this type of movement is important.

### Implications

Despite the modern hospice movement that was initiated in the UK always being known as a social movement, there is only limited evidence examining the modern hospice movement from a theoretical perspective. Focusing on the EoLC in Macao, this study has provided a new way to examine the development of EoLC by way of a social movement theory: the Framing Perspective. The findings of this study have broadened the understanding of the development of EoLC in the social movement sense. Whilst the Framing Perspective informed the consequential relationship between the diverse strategies in developing EoLC and their negative impact on EoLC, the Framing Perspective was unable to fully explain the phenomenon wherein the EoLC in Macao was developed upon initiator’s individual interpretations of the grievance. The use of the Framing Perspective has to an extent limited the understanding of the development of EoLC if and when perceived from a social movement perspective. This limitation again provides an opportunity for future research to consider other social movement theories in exploring the development of EoLC, not merely in Macao but also in other socio-cultural contexts.

## Conclusion

This study develops an understanding of the development of EoLC in the specific socio-cultural context of Macao at both the theoretical and experiential level. This research makes an important contribution in illustrating and understanding the development of the modern hospice movement in the Chinese context of Macao. Despite claims internationally that the hospice is a social movement [1, 55] there has been limited empirical and theoretical basis to support the claim. This research provides empirical evidence examining the mobilisation of the modern hospice movement from a theoretical social movement perspective. The social movement theory of the Framing Perspective [40–42] enables key facets of the development of EoLC to be explained and understood. The elements that have facilitated/inhibited the mobilisation of the social movement of the modern day hospice are valuable to understand for those seeking to develop or adapt hospice services around the globe. The lens of the social movement theory of the Framing Perspective as applied to the understanding of EoLC in the Chinese socio-cultural environment brings a unique dimension to the current understandings of the hospice development pertinent for international consideration.

## Supplementary Information

**Additional file 1.** Interview guide - English version.

## Data Availability

The audio-taped and transcribed interviews are not publicly available to protect participants’ confidentiality. Raw data may be obtained from the corresponding author on reasonable request.

## Abbreviations

HK: Hong Kong
EoL: End of Life
EoLC: End of Life Care

## References

[1] Clark D. Originating a movement: Cicely Saunders and the development of St Christopher’s Hospice, 1957–1967. Mortality. 1998;3(1):43–63.

[2] Rhymes J (1990). Hospice care in America. JAMA.

[3] Clark D, ten Have H, Janssens R. Common threads? Palliative care service developments in seven European countries. Palliative Med. 2000;14(6):479–90. 10.1191/026921600701536408 11219878

[4] Sikorska E. The hospice movement in Poland. Death Stud. 1991;15(3):309–16. 10.1080/07481189108252434 10111193

[5] Kubiak AE, Suriková M (2010). The hospice movement: the example of conflict between the process of personalized and rationalized institutionalization. Hospicové hnutie: príklad konfliktu medzi personalizovanou a racionalizovanou inštitucionalizáciou.

[6] Brown P, Flores R (2011). Making normative structures visible: The British National Health Service and the hospice movement as signifiers of compassion and hope. Acta Sociol.

[7] Bodek H (2013). Facilitating the provision of quality spiritual care in palliative care. OMEGA-J Death Dying.

[8] Lai YL, Su WH (1997). Palliative medicine and the hospice movement in Taiwan. Support Care Cancer.

[9] Wright DNM, Wood J, Lynch T, Clark D. Mapping levels of palliative care development: a global update 2011. England: Worldwide Palliative Care Alliance; 2011.

[10] Clark D, Wright M (2007). The international observatory on end of life care: a global view of palliative care development. J Pain Symptom Manage.

[11] Wright M, Hamzah E, Phungrassami T, Bausa-Claudio A. Hospice and Palliative Care in South-eastern Asia: A review of developments and challenges in Malaysia, Thailand and the Philipines. Lancaster: International Observatory on End of Life Care; 2008.

[12] Wright M, Wood J, Lynch T, Clark D (2008). Mapping Levels of Palliative Care Development: A Global View. J Pain Symptom Manage.

[13] Clark D, Graham F (2011). Evolution and change in palliative care around the world. Medicine.

[14] Luczak J, Hunter GP (2000). Hospice care in eastern Europe. Lancet.

[15] Leong RLB (2004). Palliative care in Malaysia: a decade of progress and going strong. J Pain Palliative Care Pharmacother.

[16] Nervi F, Guerrero M, Reyes MM, Nervi B, Cura A, Chávez M (2004). Symptom control and palliative care in Chile. J Pain Palliative Care Pharmacother.

[17] Nixon A (2004). Palliative care in Saudi Arabia: a brief history. J Pain Palliative Care Pharmacother.

[18] Rajagopal M, Venkateswaran C (2004). Palliative care in India: successes and limitations. J Palliative Care Pharmacother.

[19] McDermott E. Advocating hospice and palliative care: Challenges, contexts and changes. Report of the 2nd Global Summit of National Hospice and Palliative Care Associations; Worldwide Palliative Care Alliance; 2005.

[20] Bingley A, Clark D (2009). A Comparative Review of Palliative Care Development in Six Countries Represented by the Middle East Cancer Consortium (MECC). J Pain Symptom Manage.

[21] Glass AP, Chen L-K, Hwang E, Ono Y, Nahapetyan L (2010). A Cross-Cultural Comparison of Hospice Development in Japan, South Korea, and Taiwan. J Cross-Cultural Gerontol.

[22] Krongyuth P, Campbell CL, Silpasuwan P (2014). Palliative care in Thailand. Int J Palliative Nurs.

[23] Krakowiak P, Skrzypińska K, Damps-Konstańska I, Jassem E. Walls and Barriers. Polish Achievements and the Challenges of Transformation: Building a Hospice Movement in Poland. J Pain Symptom Manage. 2016;52(4):600–4. 10.1016/j.jpainsymman.2016.07.009 27524404

[24] Rhee JY, Garralda E, Torrado C, Blanco S, Ayala I, Namisango E (2017). Palliative care in Africa: a scoping review from 2005–16. Lancet Oncol.

[25] Hodges E, Read S (2018). How might organisational institutionalism support the challenges of the modern hospice?. Int J Health Plann Manage.

[26] Elsey B (1998). Hospice and palliative care as a new social movement: a case illustration from South Australia. J Palliative Care.

[27] The Printing Bureau Macao. Association of Friends of Charity of Macau Macao: The Government of Macao Special Administrative Region; 1996. Available from: http://cn.io.gov.mo/Priv/record/39.aspx. Accessed 28 Jan 2021.

[28] The Printing Bureau Macao. Macau Medical Mission Macao: The Government of Macao Special Administrative Region; 1995. Available from: http://en.io.gov.mo/Priv/record/3086.aspx. Accessed 28 Jan 2021.

[29] The Economist Intelligence Unit. The 2015 Quality of Death Index: Ranking palliative care across the world. London: The Economist Intelligence Unit; 2015. p. 15.

[30] Stake RE. The art of case study research / Robert E. Stake: Thousand Oaks, Calif. ; London : Sage, c1995.; 1995.

[31] Stake RE, Denzin NK, Lincoln YS (2000). Case Studies. Handbook of Qualitative Research.

[32] Stake RE, Denzin NK, Lincoln YS (2003). Case Studies. Strategies of Qualitative Inquiry.

[33] Wong KM, Choa M, Leong IS, Wong IL, Leong SM (2002). Cognition and Attitude of the Nurses in Kiang Wu Hospital of Macau Towards Hospice Service. Macau J Nurs.

[34] Leong SF, Chan PN, Chio IP, Lo LC, Lei SP. Student nurses’ attitude and cognition towards hospice care in Macau. Macau J Nurs. 2007;6(2):20–2.

[35] Ng WI, Ku WH (2010). Experiences of Diagnosis Disclosure of the Cancer Patients in Macau. Macau J Nurs.

[36] Bryman A. Social research methods. Oxford: Oxford university press; 2015.

[37] Twinn S (1997). An exploratory study examining the influence of translation on the validity and reliability of qualitative data in nursing research. J Adv Nurs.

[38] Regmi K, Naidoo J, Pilkington P (2010). Understanding the processes of translation and transliteration in qualitative research. Int J Qual Methods.

[39] Braun V, Clarke V (2006). Using thematic analysis in psychology. Qual Res Psychol.

[40] Snow DA, Rochford EB, Worden SK, Benford RD (1986). Frame Alignment Processes, Micromobilization, and Movement Participation. Am Sociol Rev.

[41] Snow DA, Benford RD (1988). Ideology, frame resonance, and participant mobilization. Int Soc Movement Res.

[42] Snow D, Benford R, McCammon H, Hewitt L, Fitzgerald S (2014). The Emergence, Development, and Future of the Framing Perspective: 25+ Years Since" Frame Alignment". Mobilization Int Q.

[43] Goffman E (1974). Frame analysis: An essay on the organization of experience.

[44] Benford RD, Snow DA (2000). Framing processes and social movements: An overview and assessment. Ann Rev Sociol.

[45] Denzin NK, Lincoln YS. Strategies of qualitative inquiry / Norman K. Denzin, Yvonna S. Lincoln, editors: Thousand Oaks, [Calif.] ; London : Sage, c2003. 2nd ed.; 2003.

[46] Hesse-Biber SN. Feminist Research: Exploring, Interrogating, and Transforming the Interconnections of Epistemology, Methodology, and Method. Handbook of Feminist Research. 2014:1–26.

[47] Thomas G. How to do your case study: Sage; 2021.

[48] Lancaster K (2017). Confidentiality, anonymity and power relations in elite interviewing: conducting qualitative policy research in a politicised domain. Int J Soc Res Methodol.

[49] Zhang Z, Chen M-L, Gu X-L, Liu M-H, Cheng W-W (2015). Cultural and Ethical Considerations for Cardiopulmonary Resuscitation in Chinese Patients With Cancer at the End of Life. Am J Hospice Palliative Med.

[50] Wang Z, Li Y-S, Zhao N, Yang J-J, Tu H-Y, Wu Y-L (2016). Do-not-resuscitate orders among advanced-stage Chinese lung cancer patients who died in hospital. Support Care Cancer.

[51] Hunt SA, Benford RD (1994). Identity Talk in the Peace and Justice Movement. J Contemp Ethnography.

[52] Johnston H. What is a social movement?: Cambridge, UK : Polity; 2014.

[53] Benford R (1993). Frame Disputes within the Nuclear Disarmament Movement. Soci Forces.

[54] d’Anjou L, Van Male J. Between old and new: social movements and cultural change. Mobilization Int Q. 1998;3(2):207–26.

[55] Clark D (2001). A special relationship: Cicely Saunders, the United States, and the early foundations of the modern hospice movement. Illness Crisis Loss.

