# Supplementary material for: Case study analysis of end of life care development in the Chinese cultural context of Macao: a social movement perspective
Source: BMC Palliat Care. 2021 Jul 9;20:105. doi: 10.1186/s12904-021-00807-1 (PMC8272259; doi:10.1186/s12904-021-00807-1)
Supplement: Supplementary file 1 — Additional file 1. Interview guide - English version. [file 12904_2021_807_MOESM1_ESM.docx]

Appendix 1: Interview guide - English version

Thank you for taking time to take part in this interview today. The interview today will last for approximately 45 to 60 minutes and it will be an one off interview, no other interview will be required after. You have the right not to answer any particular questions during this interview.

The entire interview will be recorded digitally and the recording is confidential, no one else except me will have access to the information. You will be kept anonymous and you will be represented by a reference number. I will not be sharing information about you or your interview with any third-party. Do you have any questions regarding this interview or the study?

Recording begins:

1. Could you tell me a bit about yourself and your professional background?
2. How and when did you become aware of EoLC?
   1. What brought you to EoLC? Is there any particular (personal/professional) experience that prompted your interest?
   2. When did you start being involved in the development of EoLC in Macao?
   3. Were there any particular reasons that lead you to develop/involve in developing EoLC?
   4. When you first started, what was the situation of EoLC at that time?
   5. How did the healthcare system/society handle the terminally ill and their families at that time?
   6. Was there any other services that you know of?
   7. What kind of support did you receive when you first started EoLC in Macao?
   8. What does EoLC (concept/ service) in Macao entail in your opinion?
   9. What was your initial goal?
3. In your opinion, what were the most important elements when you were starting/developing the EoLC in Macao?
   1. Were there any challenges in developing EoLC in Macao?
   2. What were they?
   3. What elements do you think, impact the most in the development of EoLC?
4. What was the (professional /public) reaction at the beginning?
   1. Reaction after EoLC introduction?
   2. In your opinion, how do you think the society of Macao is adjusting to EoLC after it was introduced?
5. Over the years of your involvement in EoLC in Macao, did you notice any changes? (Service/public/professional perception/understanding)
   1. Do you think the EoLC in Macao now is adequate? Do you think it needs to be improved/changed?
6. Other questions:
   1. Anything else you want to comment/ add?
   2. Would you like a copy of your interview transcription?

Recording ends.
